# Translocation of bacteria from the gut to the eggs triggers maternal transgenerational immune priming in *Tribolium castaneum*

**DOI:** 10.1098/rsbl.2015.0885

**Published:** 2015-12

**Authors:** Eileen Knorr, Henrike Schmidtberg, Derya Arslan, Linda Bingsohn, Andreas Vilcinskas

**Affiliations:** 1 Department of Bioresources, Fraunhofer Institute for Molecular Biology and Applied Ecology, Winchester Strasse 2, 35395 Giessen, Germany; 2 Institute for Insect Biotechnology, Justus-Liebig-University of Giessen, Heinrich-Buff-Ring 26-32, 35392 Giessen, Germany

**Keywords:** transgenerational immune priming, innate immunity, parental investment, fitness costs, maternal inheritance, *Tribolium castaneum*

## Abstract

Invertebrates can be primed to enhance their protection against pathogens they have encountered before. This enhanced immunity can be passed maternally or paternally to the offspring and is known as transgenerational immune priming. We challenged larvae of the red flour beetle *Tribolium castaneum* by feeding them on diets supplemented with *Escherichia coli*, *Micrococcus luteus* or *Pseudomonas entomophila*, thus mimicking natural exposure to pathogens. The oral uptake of bacteria induced immunity-related genes in the offspring, but did not affect the methylation status of the egg DNA. However, we observed the translocation of bacteria or bacterial fragments from the gut to the developing eggs via the female reproductive system. Such translocating microbial elicitors are postulated to trigger bacterial strain-specific immune responses in the offspring and provide an alternative mechanistic explanation for maternal transgenerational immune priming in coleopteran insects.

## 1. Introduction

Invertebrates can mount specific immune responses against previously encountered pathogens [1,2], although this phenomenon varies in its specificity [3,4]. The priming effect can even be transferred to the next generation to increase offspring survival following exposure to the same pathogen. This effect, known as transgenerational immune priming (TGIP), has been described for crustaceans [1], insects [5] and molluscs [6]. Both males and females can deliver information about the pathogens they have encountered to their offspring, but maternal and paternal TGIP in beetles differs in terms of specificity and the investment of resources [6–9].

The mechanisms underlying TGIP are not clearly understood. Passive mechanisms such as the transfer of antimicrobial peptides or mRNAs encoding immunity-related proteins would confer transient immunity, but full protection would require a more stable mechanism, and epigenetic modifications have been proposed. The latter include, for example, changes in DNA methylation in the parental genome caused by the first encounter with a pathogen that is transferred to the offspring as a methylation imprint in the eggs and sperm, allowing the pre-emptive activation of immunity-related genes [10,11]. Recently, a new TGIP mechanism was identified in the greater wax moth *Galleria mellonella* involving the transfer of ingested bacteria from the maternal gut to the eggs [12].

To determine whether similar TGIP mechanisms may be involved in *T. castaneum*, an insect that is now established as a model for bacterial oral infections [13] and in which both maternal and paternal TGIP have already been confirmed [14], we investigated the transfer of bacterial particles ingested by female beetles and the methylation status of DNA in the offspring.

## 2. Material and methods

### (a) Insect rearing and treatment

Wild-type *Tribolium castaneum* San Bernardino beetles were reared as described elsewhere [15]. Neonate larvae were fed until the adult stage on diets supplemented with 0.3% lyophilized *Escherichia coli, Micrococcus luteus* or *Pseudomonas entomophila*. A non-supplemented diet was used as a control treatment. The adults were removed at 10 days old and transferred to the control diet. Eggs were collected by sieving after 24 h.

### (b) RNA isolation and quantitative real-time PCR

Total RNA was extracted from pooled eggs (50 mg, three biological replicates) using Direct-zol™ RNA MiniPrep (Zymo Research, Irvine, CA) according to the manufacturer's instructions. The quality and quantity of RNA were determined using a Nanodrop ND-1000 spectrophotometer (NanoDrop Technologies Inc., Wilmington, DE). We reverse transcribed 50 ng total RNA using the first-strand cDNA synthesis kit (Thermo Fisher Scientific, Rockford, IL) and carried out quantitative real-time PCR using the Power SYBR^®^ Green PCR master mix (Thermo Fisher Scientific) with a StepOne plus real-time PCR system (Thermo Fisher Scientific). Primers were designed using primer3 (http://bioinfo.ut.ee/primer3-0.4.0/) and were purchased from Sigma-Aldrich (St Louis, MO).

Three biological replications, each with two technical replications and no template controls, were run in parallel. Relative gene expression levels were calculated using the 2^−ΔΔCT^ method [16] with the ribosomal protein gene *Rps3* as a reference. Statistical analysis was carried out using SigmaPlot v. 12.0 (Systat Software Inc., San Jose, CA). Significant differences between groups of parametric data were determined by one-way analysis of variance (ANOVA) with a subsequent Holm–Sidak test (*p* < 0.05). Non-parametric data were analysed by ANOVA on-ranks with a subsequent Tukey's test.

### (c) Methylation assay

Eggs (50 mg, three biological replicates) from adults (5–7 days old) raised on white flour supplemented with bacteria (see above) were collected for DNA isolation using the ZR Tissue and Insect MicroPrep kit (ZymoResearch), and the global DNA methylation status was determined using the colorimetric MethylFlash methylated DNA quantification kit (Epigentek, Farmingdale, NY) according to the manufacturer's instructions. The absolute amount of methylated DNA was calculated from 100 ng total DNA using a standard curve.

### (d) Analysis of fluorescent BioParticles^®^

Animals were fed on an artificial agar-based diet containing 5% whole wheat flour, 15% yeast, 3% agar and 0.4% methyl hydroxybenzoate. The artificial diet was supplemented with *E. coli* (K-12 strain) cells (3 × 10^6^ *E. coli* µl^−1^) conjugated with 25 µl Texas Red^®^ BioParticles^®^ (Thermo Fisher Scientific) per 1 ml agar, suspended at a concentration of 10 mg ml^−1^ in 10 mM PBS. Decapitated last-instar larvae and adult females, as well as dissected reproductive tissue and ovipositioned eggs, were embedded in Tissue-Tek^®^ OCT™ (Sakura^®^ Finetek). Samples were frozen in liquid nitrogen and stored at –80°C. A cryostat microtome CM 1850 (Leica Microsystems) was used to prepare 10 µm sections at –20°C and these were mounted with Fluoromount-GTM (Southern Biotech) and observed under a DM5000 B fluorescence microscope (Leica; see the electronic supplementary material).

## 3. Results

### (a) Expression of immunity- and stress-related genes

**Figure 1.**
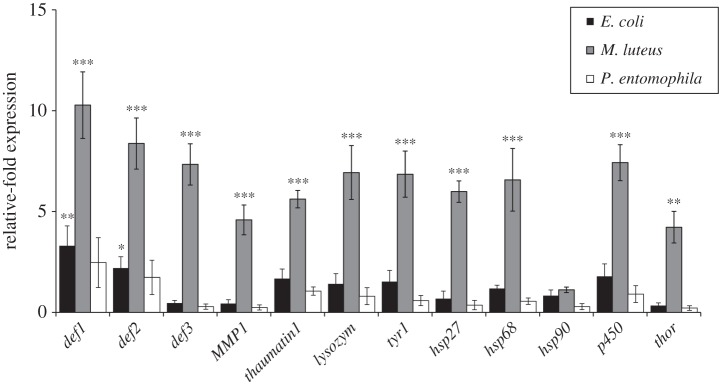
Relative expression levels of immunity- and stress-related genes in naive *Tribolium* castaneum eggs. RNA was isolated from pooled 50 mg samples of eggs laid by parents fed on bacterial diets. Expression levels are presented relative to eggs from non-supplemented diet and normalized against the endogenous housekeeping gene *Rps3*. The data represent means (±s.d.) of three independent biological replicates (one-way ANOVA, Holm–Sidak, ***p < 0.001).

We compared the expression profiles of immunity- and stress-related genes in eggs laid by naive parents, and adults reared on diets supplemented with *E. coli*, *M. luteus* or *P. entomophila.* Quantitative real-time PCR data for seven immunity-related genes and five stress-related genes (encoding cytochrome p450, Thor and heat shock proteins (hsp) 27, 68 and 90) showed that the offspring of beetles ingesting *M. luteus* displayed by far the highest expression levels. With the exception of *hsp90*, all genes were induced by more than fourfold compared with the control treatment (figure 1). *Defensin 1* was induced most strongly, with expression levels increasing by more than 10-fold (mean fold change = 10.27). Contamination of the larval diet with *E. coli* induced the expression of *defensin 1* (mean fold change = 3.31) and *defensin 2* (mean fold change = 2.2) in the eggs, whereas genes encoding *thaumatin* 1, *p450*, *lysozyme* and *polyphenoloxidase* were only marginally upregulated. Surprisingly, the oral uptake of *P. entomophila* had a much lower impact on gene expression, resulting in the slight induction of *defensin 1* (mean fold change = 2.47).

### (b) Maternal transfer of bacteria

The underlying physiological mechanisms of TGIP are not well understood, and several hypotheses have been proposed to explain how such information might be transferred to the offspring. One hypothesis involves the epigenetic modification of germline DNA [17]. We therefore analysed the global DNA methylation status of eggs laid by parents ingesting diets supplemented with *P. entomophila*, *E. coli* or *M. luteus*, compared with DNA from eggs laid by naive parents. We found no significant differences among the treatments. The average level of global methylation was approximately 10% (10.17% ± 0.75) under all treatment regimens (electronic supplementary material, figure S1).

**Figure 2.**
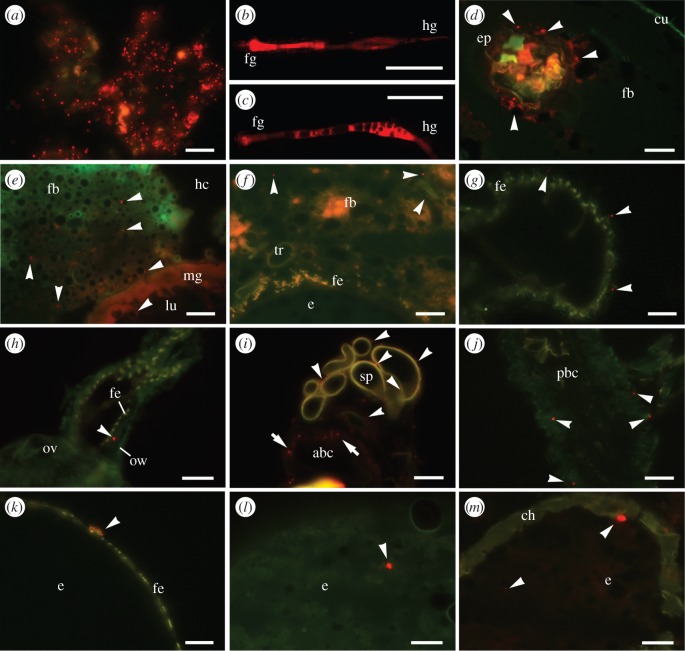
Analysis of the maternal transfer of fluorescent bacteria (BioParticles®). (*a*) Artificial diet mixed with BioParticles^®^ (scale bar, 50 µm). (*b,c*) BioParticles^®^ in the dissected larval gut after the ingestion of the artificial diet, show (*b*) the foregut and midgut region and (*c*) the hindgut (scale bars, 1 mm). (*d*) BioParticles^®^ beneath the cuticle of the larval foregut (scale bar, 100 µm). (*e*) Larval midgut containing translocated BioParticles^®^ in the lumen and surrounding fat body cells (scale bar, 50 µm). (*f*) Female genital region with BioParticles^®^ attached to the fat body (scale bar, 50 µm). (*g,h*) BioParticles^®^ between the ovariole wall and the follicular epithelium of eggs in (*g*) the proximal region (scale bar, 50 µm) and (*h*) the distal region close to the lateral oviduct (scale bar, 150 µm). (*i,j*) BioParticles^®^ associated with (i) spermatheca and the anterior bursa copulatrix, and (*j*) the posterior bursa copulatrix (scale bars, 50 µm). (*k,l*) BioParticles^®^ attached to (*k*) the follicular epithelium (scale bar, 50 µm) and (*l*) incorporated into the yolk of dissected eggs (scale bar, 150 µm). (*m*) Ovipositioned egg containing BioParicles^®^ (scale bar, 150 µm). Further details are provided in the electronic supplementary material. Arrowheads indicate fluorescent BioParticles^®^ (red spots). abc, anterior bursa copulatrix; ch, chorion; cu, cuticle; e, egg; ep, epithelium; fb, fatbody; fe, follicular epithelium; fg, foregut; hc, haemocoel; hg, hindgut; lu midgut lumen; mg, midgut; ov, oviduct, ow ovariole wall; pbc posterior bursa copulatrix; sp, spermatheca; tr, tracheole.

We investigated whether ingested bacteria are translocated from the gut into the developing eggs in *T. castaneum* by determining the fate of non-viable *E. coli* bioparticles after larval ingestion (figure 2). We established an artificial feeding assay to administer high doses of bioparticles to the larvae (electronic supplementary material, figures S2 and S3). Cryosections of last-instar larvae confirmed the translocation of fluorescent bacterial particles from the midgut epithelium into the surrounding fat body cells in the haemocoel following oral uptake (figure 2*e* and electronic supplementary material, figure S4). The genital regions of adult females fed on a diet supplemented with bacteria also contained bacterial particles attached to the fat body (figure 2*f*), and particles were also detected between the ovariole wall and the follicular epithelium of the developing eggs (figure 2*k–l* and electronic supplementary material, figure S5). Ultimately, we confirmed the presence of bacterial particles in the ovipositioned eggs (figure 2*m* and electronic supplementary material, figure S6).

## 4. Discussion

We investigated the processes underlying maternal TGIP in *T. castaneum*, focusing on two proposed mechanisms: the transfer of ingested bacterial particles from the larval gut to the eggs of adult female beetles and the methylation status of the maternal and offspring DNA. We found that the oral administration of bacteria to *T. castaneum* larvae was sufficient to induce an immune response in the next generation. Similar TGIP effects have been observed in lepidopteran species ingesting a diet supplemented with bacteria [12] and in the offspring of mealworms (*Tenebrio molitor*) injected with bacterial lipopolysaccharides [18]. The gene expression profiles in the eggs *T. castaneum* larvae fed with contaminated diet suggested that the immune response differs between Gram-positive and Gram-negative bacteria.

The physiological processes underlying TGIP remain elusive. Microbial pathogens can modulate host epigenetic regulatory factors such as the acetylation or deacetylation of histones and the expression of miRNAs, suggesting that transgenerational inheritance may be associated with epigenetic mechanisms such as DNA methylation [11,19]. However, *T. castaneum* larvae reared on a bacteria-supplemented diet did not transmit changes in the overall level of DNA methylation to the next generation, but this does rule out the possibility that differences in DNA methylation pattern may pass to the offspring. Further research is necessary to determine whether other epigenetic mechanisms such as histone acetylation are involved.

We therefore monitored the fate of bacterial particles orally administered to *T. castaneum* larvae and found that they crossed the gut epithelium and were translocated into the developing egg. Such a transfer of bacteria to the germline necessitates the protection of the eggs against pathogens. Indeed, the extraembryonic serosa of *T. castaneum* eggs is described as a frontier epithelium that expresses nearly 90% of the immunity-related genes in the egg genome, providing a full range of immune responses [20]. Such an investment in the immune competence of the serosa makes sense if bacteria from the gut can translocate into the developing eggs. Indeed, a recent study shows that the egg yolk protein vitellogenin is involved in the internalization of bacteria during oogenesis [21]. Our data provide a plausible explanation for maternal strain-specific TGIP in the model beetle *T. castaneum* and confirm the mechanism identified in lepidopteran species, suggesting that it may be a general strategy used by diverse insects. However, the maternal transfer of bacterial fragments does not explain paternal TGIP in *T. castaneum* [8], and the latter will therefore be addressed in our future studies.

## Supplementary Material

Electronic Supplementary Material

## Data accessibility

Additional data and control experiments for the microscopic studies are accessible in the electronic supplementary material.
