# Supplementary material for: Translocation of bacteria from the gut to the eggs triggers maternal transgenerational immune priming in Tribolium castaneum
Source: Biol Lett. 2015 Dec;11(12):20150885. doi: 10.1098/rsbl.2015.0885 (PMC4707703; doi:10.1098/rsbl.2015.0885)
Supplement: Electronic Supplementary Material [file rsbl20150885supp1.pdf]

Electronic Supplementary Material

Biology Letters

Knorr, E, Schmidtberg, H, Arslan, D, Bingsohn, L, Vilcinskas. Translocation of bacteria from the gut to the eggs triggers maternal transgenerational immune priming in *Tribolium castaneum*.

Supplementary Materials and Methods

(a) Methylation assay

Freshly laid eggs were transferred to whole wheat flour containing 5% (w/w) yeast supplemented with 0.3% (w/w) *P. entomophila*, *E. coli*, *M. luteus* or a mixture of all three. Pooled eggs (50 mg, three biological replicates) from adults (5-7 days old) were collected for DNA isolation using the ZR Tissue & Insect MicroPrep kit (Zymo Research, Irvine, California, USA) and the global DNA methylation status was determined using the colorimetric MethylFlash Methylated DNA Quantification kit (Epigentek, Farmingdale, New York, USA) with 100 ng input DNA according to the manufacturer's instructions. A standard curve was performed generated, and the amount and percentage of 5-methylcytidine in the total DNA was calculated as described by the manufacturer.

(b) Analysis of fluorescent BioParticles®

Artificial feeding assay

An artificial feeding assay based on agar was developed to ensure the equipartition of the *E. coli* BioParticles® conjugated with Texas Red® (Thermo Fisher Scientific, Rockford, Illinois, USA). All working steps were carried out in darkness to protect the fluorophores. The BioParticles® were dissolved at a concentration of 10 mg/ml in 10 mM PBS to make a stock solution corresponding to 3 x 10^6 bacteria per µl. The suspension was incubated for 15 min in an ultrasonic bath. Larvae were fed on a basal diet of 5% whole wheat flour, 15% yeast and 3% agar supplemented with 0.4% methyl hydroxybenzoate to prevent fungal growth. The ingredients were mixed together, autoclaved and poured into glass Petri dishes. The agar was dried for 4 h at 32°C and 500 µl of the BioParticles® solution was then plated on the agar. The plates were packed in aluminium foil and dried for an additional 4 h at 32°C. The same agar mixture without BioParticles® was used as a control diet.

Microscopy

Neonates were fed until adulthood on an artificial diet mixed with Bioparticles® (see above) or without the Bioparticles® as a control. For fluorescence microscopy, fifth-instar larvae and adult females (~30 individuals per developmental stage and treatment) were decapitated, embedded in Tissue-Tek® OCT™ (Sakura, Finetek, Germany) and frozen at –80°C. Dissected female reproductive organs and ovipositioned eggs were processed in the same manner. Sections (10 µm) were prepared with a Leica CM1850 cryotome, mounted with Fluoromount-G™ (Southern Biotech, Birmingham, Alabama, USA) and examined using a Leica DM5000 B fluorescence-microscope (Leica Microsystems, Wetzlar, Germany). Fluorescence photomicrographs were acquired with overlays of N3 and L5 fluorescence filters (Leica) to optimize the fluorescence visualization. Corresponding micrographs were also prepared with control filters and in bright field.
The tissues containing Bioparticles® were also analyzed as semi-thin sections. *T. castaneum* larvae and adults were dissected in cold 0.1 M phosphate buffer (pH 7.4), fixed in 2.5% glutaraldehyde and 2% paraformaldehyde in 0.1 M phosphate buffer (pH 7.4) for 2 h and post-fixed in 1% OsO4 in the same buffer for 1 h at room temperature. The material was dehydrated through a graded ethanol series and embedded in Durcupan (Sigma-Aldrich). Sections (1 µm) were prepared on a Reichert Om/U2 ultramicrotome and stained with toluidine blue.

absolute							
	naïve	*P. entomophila*	*E. coli*	*M. luteus*	*Bacteria mix*		
1	7,82	10,04	13,21	6,82	9,80		
2	6,01	11,78	10,87	16,16	9,34		
3	14,49	9,99	8,38	9,17	8,66		
mean	9,44	10,60	10,82	10,72	9,27	10,169967	overall average
SD	4,47	1,02	2,41	4,86	0,57	0,74902883	SD
SE	2,58	0,59	1,39	2,81	0,33		

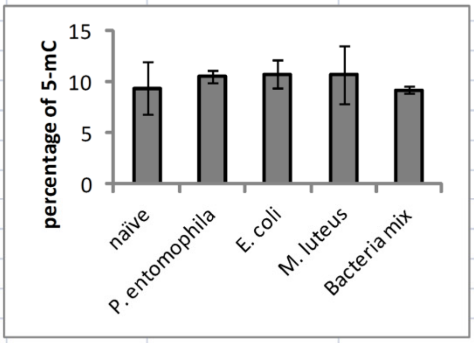

Supplementary Figure S1. Status of global DNA methylation in eggs of *T. castaneum*. Methylated DNA status of *Tribolium* eggs was detected with a calorimetric assay using the absolute quantification as described in manufacturer's instructions. Methylation in the eggs laid by parents fed on bacterial diets compared to naïve. Results were calculated from the mean values of three independent biological replicates ± SE.

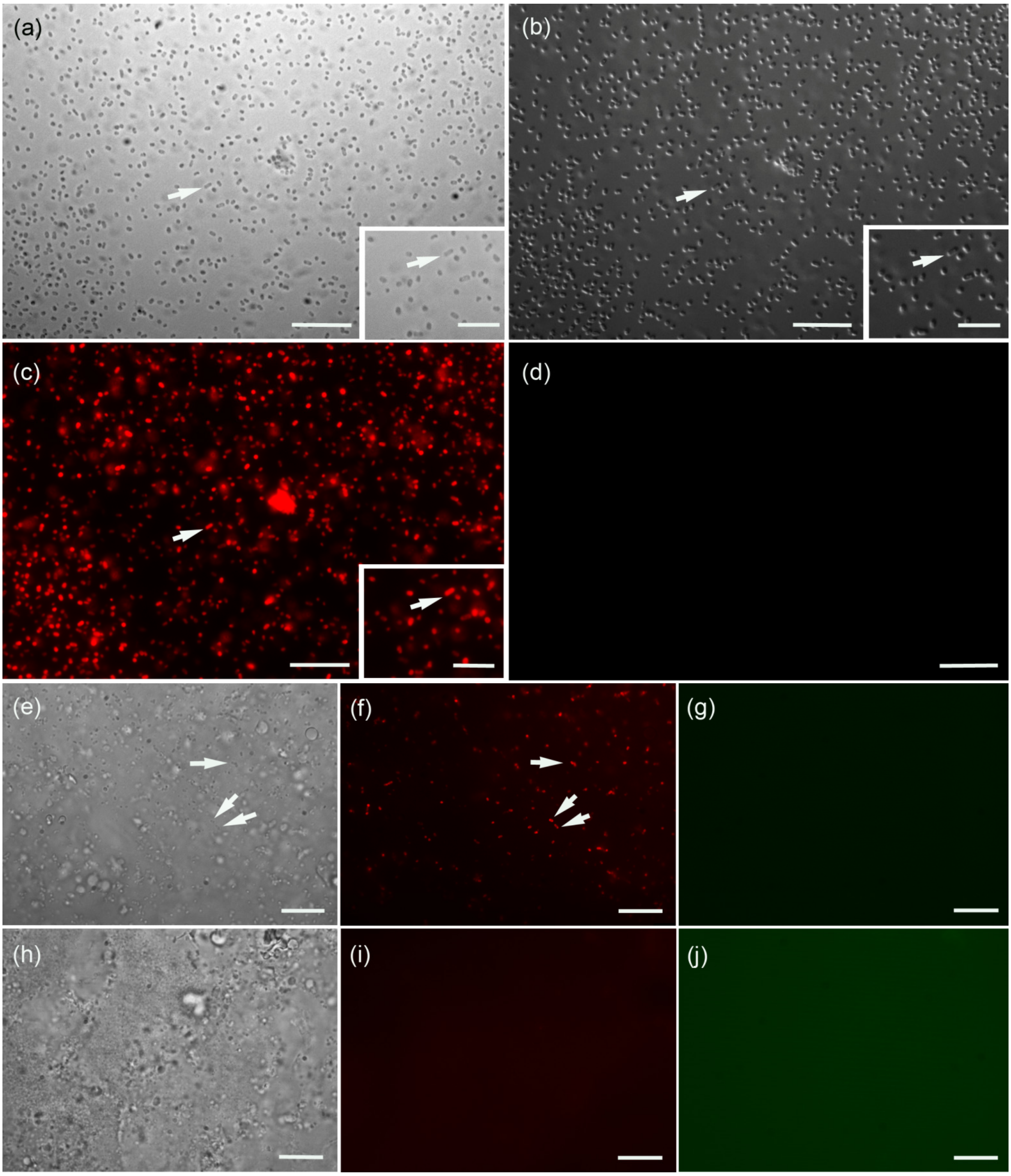

Supplementary Figure S2. Wavelength specificity of Texas Red® conjugated *E. coli* BioParticles®. According to manufacturer's data, the fluorescent Texas Red® particles are bound to the surface of the bacterial cells. To characterize the wavelength specificity of the Texas Red®-conjugates we used different filtercubes. In the first experiment (a-d), we suspended the BioParticles® in phosphate buffered saline (PBS). Under brightfield microscopy (a) and differential interference contrast microscopy (b) single bacterial cells can be differentiated. The arrows indicate the appropriate identical bacterial cell. (c) The application of the fluorescence filtercube N3 (Leica, wavelength BP 546/612) stimulates the emission of Texas Red® (red spots), whereas the application of filtercube L5 (Leica, wavelength BP 480/540) functions as control filter and no fluorescence is noticeable (d); scale bars 20 µm. Insets are magnifications of the respective same areas with scale bars 10 µm. For the feeding assays, we mixed the agar-based diet for the *Tribolium* larvae with fluorescent bacteria (e-g). The latter were detectable under brightfield microscopy (e, arrows) and fluorescence microscopy using filtercube N3 with red spots indicating fluorescent bacteria (f). The arrows point to the respective identical BioParticles; scale bars 20 µm. As control experiment, we fed *Tribolium* larvae with agar-based diet lacking BioParticles® (h-j) and therefore no bacteria could be detected neither under brightfield microscopy (h) nor under fluorescence microscopy with (i) filtercube N3. (g, j) Control filtercube L5 of the respective areas; scale bars 20 µm.

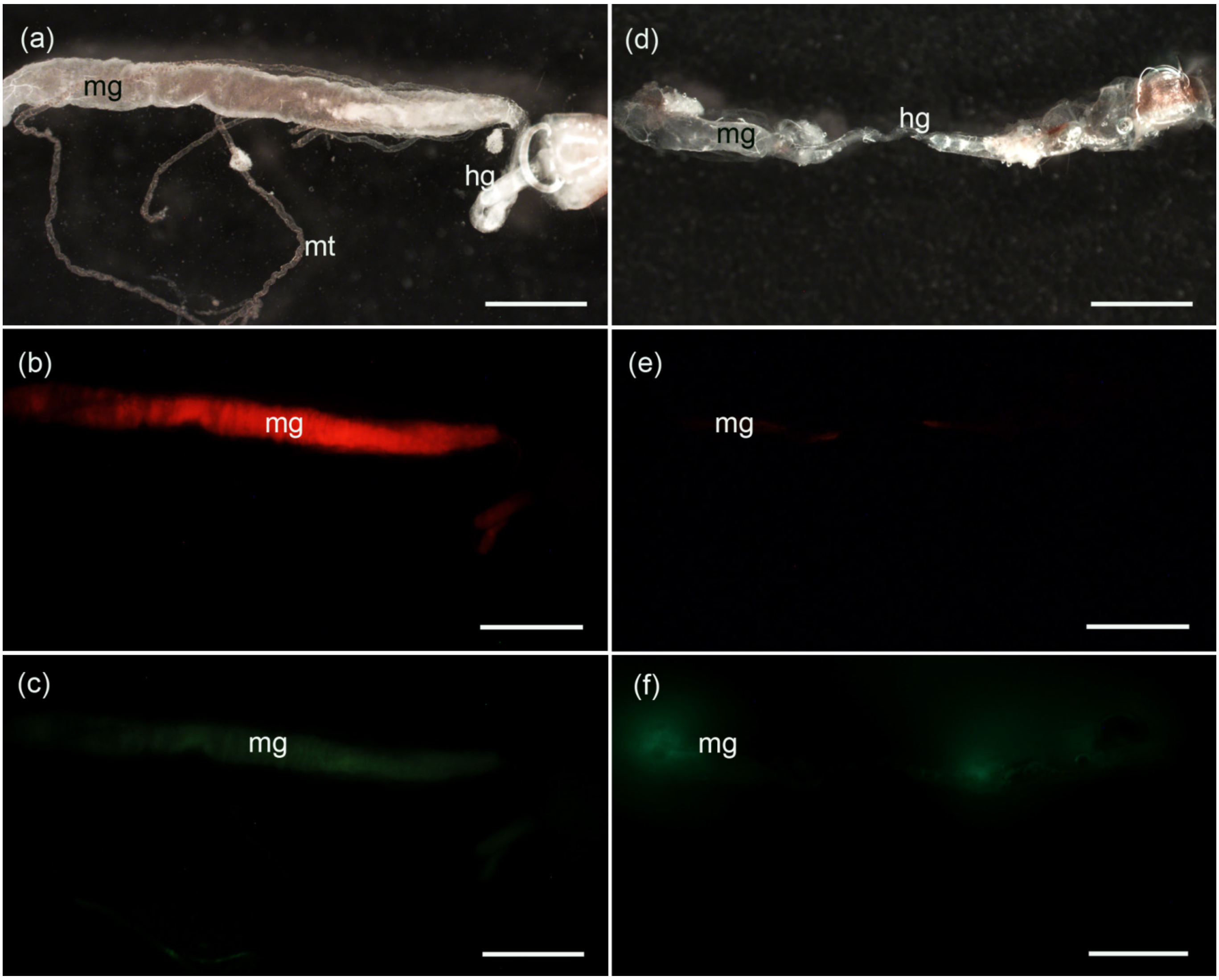

Supplementary Figure S3. Dissected guts of last larval stages of *T. castaneum.* To visualize the uptake of fluorescent bacteria in the gut, we dissected guts of larva fed with (a-c) artificial diet mixed with fluorescent bacteria and (d-f) control larva with artificial diet exclusive of BioParticles®. (a) A dissected gut under brightfield microscopy and fluorescence microscopy with (b) filtercube N3 illustrates red fluorescence of gut lumen after uptake of BioParticles®. In contrast, the gut of the control larva (d) shows no red fluorescence (e) with filtercube N3, because no BioParticles® were ingested. (c, f) Fluorescence microscopy with control filtercube L5 with slightly green auto-fluorescence of the gut tissue. Scale bars 500 µm.

Abbreviations: hg, hindgut; mg, midgut; mt, Malpighian tubules.

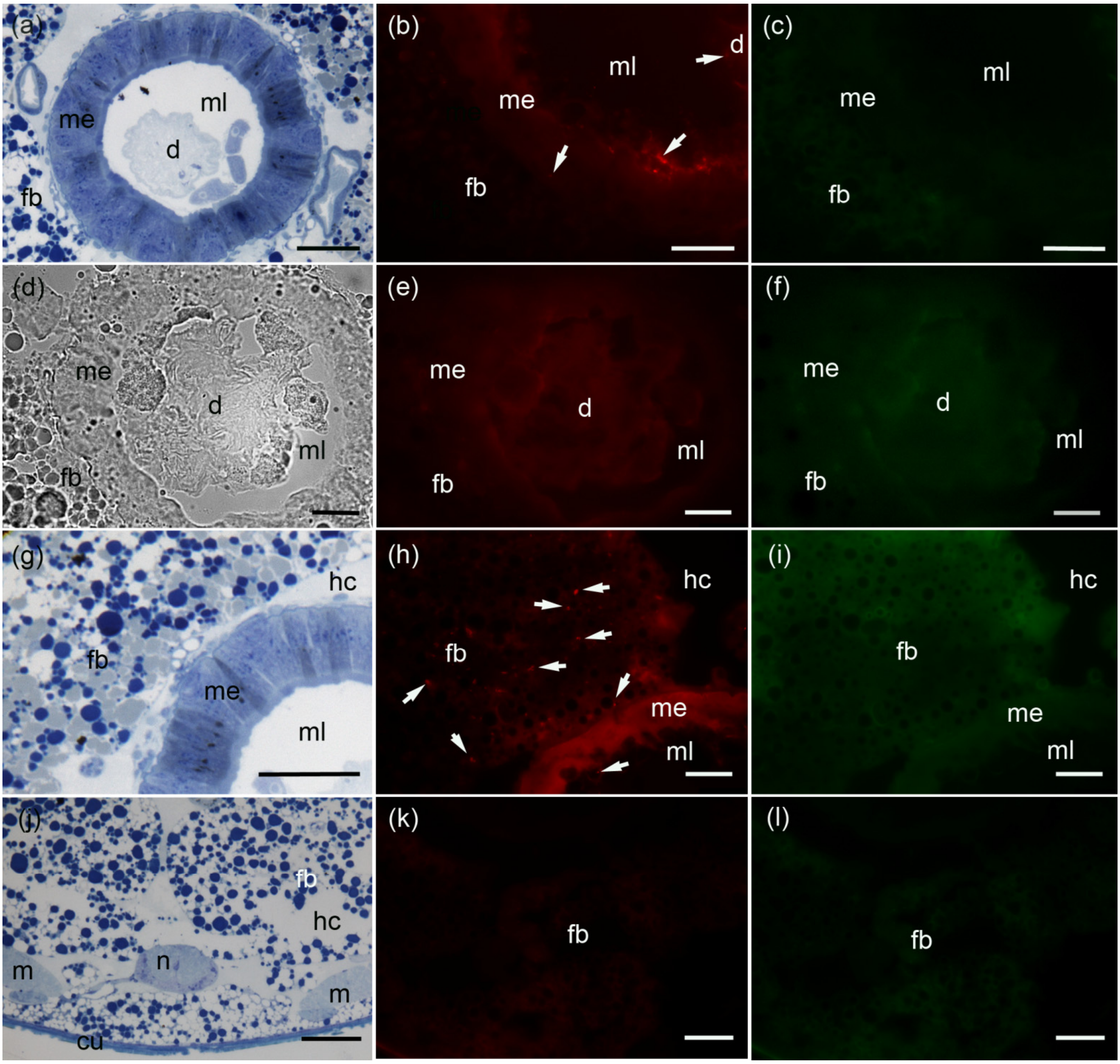

Supplementary Figure S4. Combination of fluorescence and brightfield microscopy for localization of Texas Red® conjugated *E. coli* BioParticles® in gut and fatbody of treated and control larvae of *T. castaneum*. For better orientation and determination of different tissues, semithin sections of relevant regions have been made in areas with fluorescent bacteria. (a-c) Micrographs of sections of the midgut region of treated larvae fed with BioParticles®. (a) The larval midgut is surrounded by fatbody tissue. The midgut epithelium encircles the midgut lumen which is filled with uptaken diet. Fluorescence microscopy of cryosections of the same area with (b) application of filtercube N3 reveals fluorescent bacteria in the midgut lumen and the uptake into the midgut epithelium (arrows). (d-f) In the cryosections of the midgut region of control larvae fed without BioParticles® fluorescent bacteria are neither found under (d) brightfield nor under fluorescence microscopy with application of (e) filtercube N3 of the same area. (g-i) Larval fatbody of individuals fed with BioParticles®. (g) Semithin section of the fatbody tissue in close neighbourhood to midgut epithelium. Under fluorescence microscopy with application of filtercube N3 (h), we detect in cryosections of the respective area red spots in the fatbody tissue indicating that BioParticles® are translocated from the midgut lumen into the midgut epithelium to the surrounding hemocoel and then uptaken into the fatbody tissue (arrows). (j-l) Fatbody tissue of control individuals. (j) Semithin sections and fluorescence microscopy with application of (k) filtercube N3 of the same area reveal no fluorescent bacteria. (c,f,I,l) Applications of control filtercube L5 in the respective cryosections. Scale bars 50 µm.

Abbreviations: cu, cuticle; d, diet; fb, fatbody; hc, hemocoel; m, muscle; me, midgut epithelium; ml, midgut lumen; n, nervous tissue.

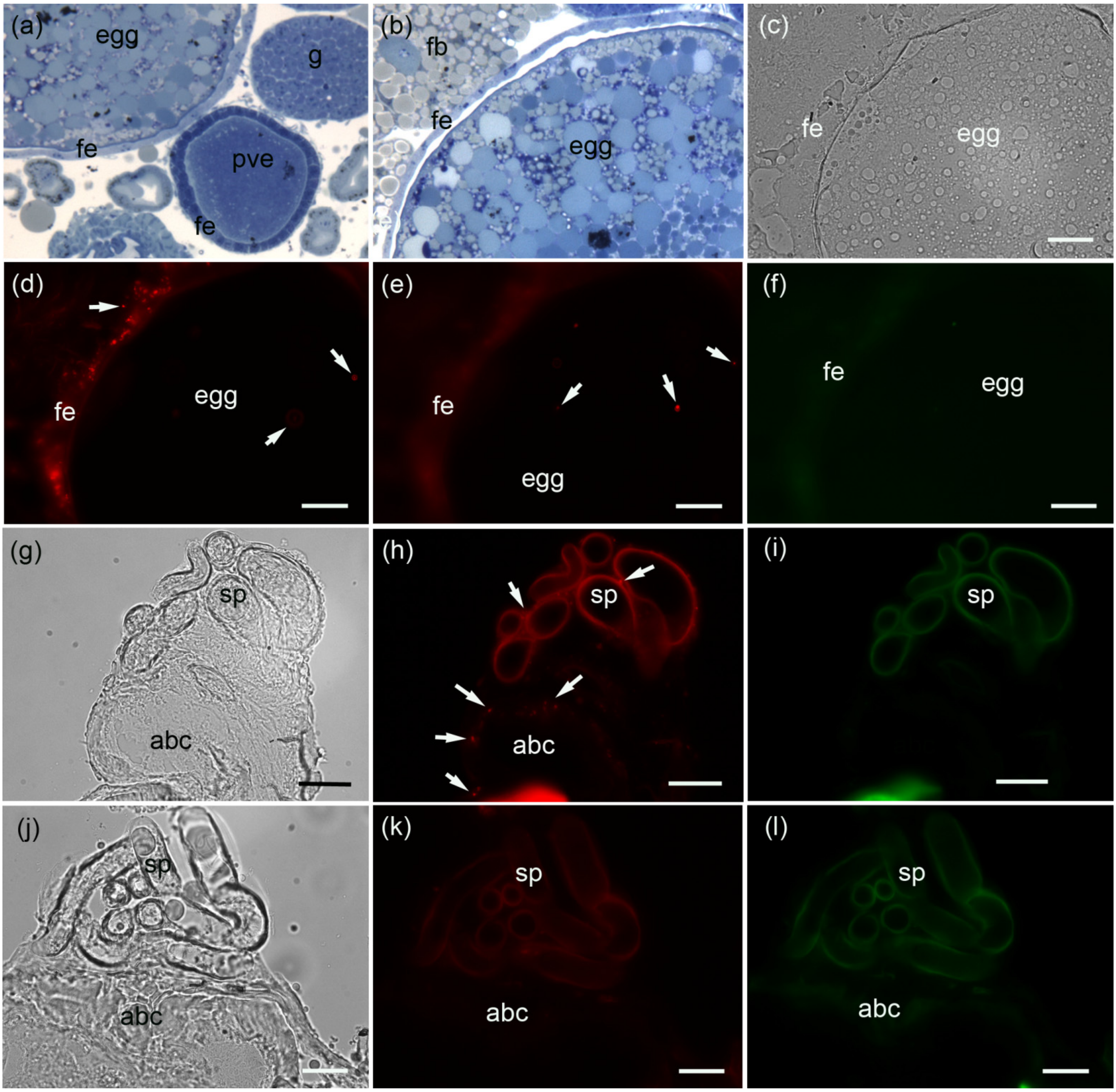

Supplementary Figure S5. Combination of fluorescence and brightfield microscopy for localization of Texas Red® conjugated *E. coli* BioParticles® in reproductive tissues, oocytes and eggs of adult treated and control females of *T. castaneum*. (a) Semithin section of the ovary with germarium, pre-vitellogenin oocyte and vitellogenin oocyte, and (b) magnification of a vitellogenin oocyte with large yolk granules. (c-f) Cryosections of a vitellogenin oocyte in the ovary of an adult female. (c) Brightfield microscopy and (d, f) fluorescence microscopy of the same cryosection with N3 filtercube. (d) Arrows point to the fluorescence bacteria close to the follicle epithelium and inside the egg. This indicates that already in the ovary bacteria can be translocated into the oocytes. (e) In another focus level of the same cryosection further spots of BioParticles® appear (arrows). (g-i) Cryosections of spermatheca and anterior bursa copulatrix of a treated female fed with BioParticles® under (g) brightfield and (h, i) fluorescence microscopy. (h) Fluorescent bacteria are detectable with filtercube N3 in the spematheca and the anterior bursa copulatrix (arrows). (j-l) Cryosections of a control female which was fed with artificial diet exclusive of BioParticles®. (j) Brightfield and (k) fluorescence microscopy with N3 filtercube of the same cryosection show no fluorescent bacteria. (f, I, j) Control filtercube L5 in respective cryosections. Scale bars 50 µm.

Abbreviations: abc, anterior bursa copulatrix; egg, oocyte/egg; fb, fatbody tissue; fe, follicle epithelium; g, germarium; pve, pre-vitellogenin oocyte/egg; sp, spermatheca.

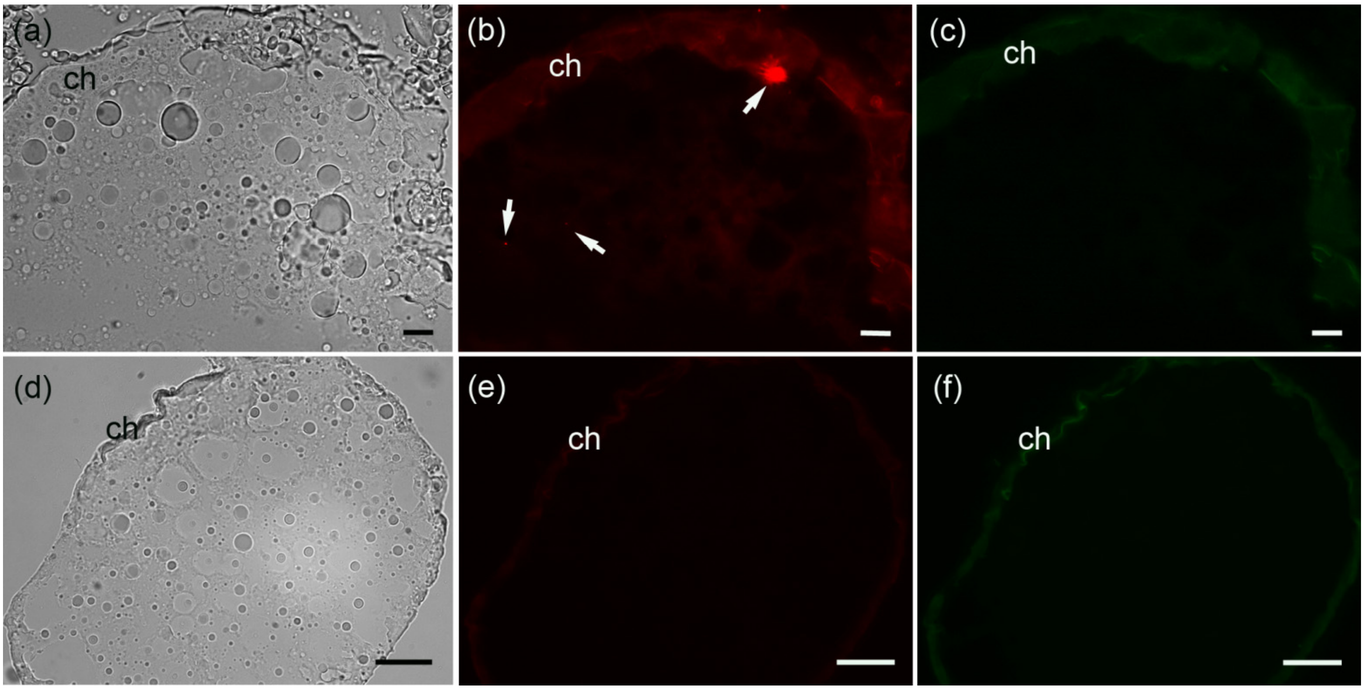

Supplementary Figure S6. Combination of fluorescence and brightfield microscopy for localization of Texas Red® conjugated *E. coli* BioParticles® of ovipositioned eggs of treated and control females of *T. castaneum*. (a-c) Cryosection of an ovipositioned egg of a female fed with fluorescent bacteria. (a) Brightfield microscopy illustrates the large yolk granules in the egg which is enveloped by the chorion. (b) BioParticles® are visible under fluorescence microscopy with filtercube N3 in the ovipositioned egg (arrows). (d-f) Cryosections of an ovipositioned egg of a control female under (d) brightfield and (e) fluorescence microscopy showing no fluorescent bacteria. (c, f) Control filtercube L5 in respective cryosections. Scale bars 50 µm.
Abbreviation: ch, chorion.
